# Leveraging speech and artificial intelligence to screen for early Alzheimer’s disease and amyloid beta positivity

**DOI:** 10.1093/braincomms/fcac231

**Published:** 2022-10-14

**Authors:** Emil Fristed, Caroline Skirrow, Marton Meszaros, Raphael Lenain, Udeepa Meepegama, Kathryn V Papp, Michael Ropacki, Jack Weston

**Affiliations:** Novoic Ltd, London, N1 7EU, UK; Novoic Ltd, London, N1 7EU, UK; Novoic Ltd, London, N1 7EU, UK; Novoic Ltd, London, N1 7EU, UK; Novoic Ltd, London, N1 7EU, UK; Center for Alzheimer Research and Treatment, Department of Neurology, Brigham and Women's Hospital, Harvard Medical School, Boston, Massachusetts, 02115, USA; Department of Neurology, Massachusetts General Hospital, Harvard Medical School, Boston, Massachusetts, 02114, USA; Strategic Global Research & Development, Temecula, California, 94019, USA; Novoic Ltd, London, N1 7EU, UK

**Keywords:** Alzheimer's disease, MCI (mild cognitive impairment), speech, artificial intelligence, machine learning

## Abstract

Early detection of Alzheimer’s disease is required to identify patients suitable for disease-modifying medications and to improve access to non-pharmacological preventative interventions. Prior research shows detectable changes in speech in Alzheimer’s dementia and its clinical precursors. The current study assesses whether a fully automated speech-based artificial intelligence system can detect cognitive impairment and amyloid beta positivity, which characterize early stages of Alzheimer’s disease. Two hundred participants (age 54–85, mean 70.6; 114 female, 86 male) from sister studies in the UK (NCT04828122) and the USA (NCT04928976), completed the same assessments and were combined in the current analyses. Participants were recruited from prior clinical trials where amyloid beta status (97 amyloid positive, 103 amyloid negative, as established via PET or CSF test) and clinical diagnostic status was known (94 cognitively unimpaired, 106 with mild cognitive impairment or mild Alzheimer’s disease). The automatic story recall task was administered during supervised in-person or telemedicine assessments, where participants were asked to recall stories immediately and after a brief delay. An artificial intelligence text-pair evaluation model produced vector-based outputs from the original story text and recorded and transcribed participant recalls, quantifying differences between them. Vector-based representations were fed into logistic regression models, trained with tournament leave-pair-out cross-validation analysis to predict amyloid beta status (primary endpoint), mild cognitive impairment and amyloid beta status in diagnostic subgroups (secondary endpoints). Predictions were assessed by the area under the receiver operating characteristic curve for the test result in comparison with reference standards (diagnostic and amyloid status). Simulation analysis evaluated two potential benefits of speech-based screening: (i) mild cognitive impairment screening in primary care compared with the Mini-Mental State Exam, and (ii) pre-screening prior to PET scanning when identifying an amyloid positive sample. Speech-based screening predicted amyloid beta positivity (area under the curve = 0.77) and mild cognitive impairment or mild Alzheimer’s disease (area under the curve = 0.83) in the full sample, and predicted amyloid beta in subsamples (mild cognitive impairment or mild Alzheimer’s disease: area under the curve = 0.82; cognitively unimpaired: area under the curve = 0.71). Simulation analyses indicated that in primary care, speech-based screening could modestly improve detection of mild cognitive impairment (+8.5%), while reducing false positives (−59.1%). Furthermore, speech-based amyloid pre-screening was estimated to reduce the number of PET scans required by 35.3% and 35.5% in individuals with mild cognitive impairment and cognitively unimpaired individuals, respectively. Speech-based assessment offers accessible and scalable screening for mild cognitive impairment and amyloid beta positivity.

## Teaser

Fristed *et al*. report that early changes in speech in Alzheimer’s disease can be leveraged using a fully automated artificial intelligence system to screen for early clinical impairment and amyloid beta positivity. Speech-based testing offers accessible and scalable screening for mild cognitive impairment and for recruitment into amyloid-targeting trials.

## Graphical Abstract

**Graphical Abstract:**
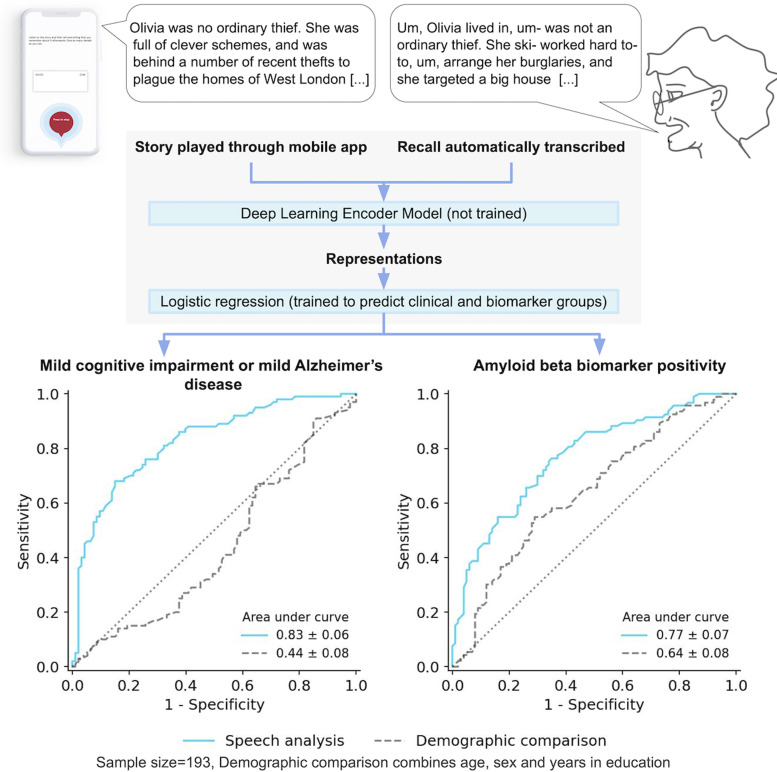

## Introduction

Alzheimer’s disease is not routinely screened for in clinical practice.^1^ Instead it is most commonly tested for when patients present with cognitive complaints, or after cognitive impairment interferes with daily functioning. Research indicates that half of individuals aged 65+ with dementia are missed from primary care dementia registers, which suggests that around 50% of cases remain undiagnosed even at the more advanced stages of Alzheimer’s disease.^2^

Alzheimer’s disease is characterized by changes in the brain including accumulation of amyloid beta (Aβ) neuritic plaques, aggregated tau neurofibrillary tangles and neurodegeneration, often beginning decades before routine diagnosis.^3^ Pathologic changes are typically tracked initially by more subtle and later by more overt cognitive and clinical symptoms and impairments.^4^

Episodic memory, commonly assessed using story recall tasks, is impaired in Alzheimer’s dementia.^5^ Story recall differentiates individuals with mild cognitive impairment (MCI; an earlier stage of the disease), from those that are cognitively unimpaired (CU)^6^ and is commonly used for screening into Alzheimer’s disease trials.^7^ Story recall is typically scored via comparison of the recalled information units with the story source, allowing for paraphrastic variation.^8^ More granular changes in story recall, such as a reduction in the recall of proper nouns^9^ and an effect of the serial position of elements recalled,^10^ have been associated with Aβ positivity in CU individuals.

There is a growing interest in speech and language data that can be collected on ubiquitous digital devices and in everyday situations or healthcare settings. Speech is functionally important and naturalistic, and is commonly elicited in cognitive tasks. Speech can be separated into linguistic (such as semantic content, syntactic complexity, repetitions) and prosodic patterns (relating to intonation and rhythm); which may be altered both in MCI and Alzheimer’s disease as shown by recent meta-analyses.^11,12^

Much of the completed speech research to date in Alzheimer’s disease has been carried out on a small number of cohorts with openly available datasets, such as the DementiaBank Pitt corpus,^13^ and the Alzheimer’s Dementia Recognition through Spontaneous Speech (ADReSS) Challenge cohort,^14^ which contains recorded picture descriptions from a cohort with probable Alzhiemer’s dementia. Notable limitations of these datasets include the small sample sizes under investigation, with participants without biomarker confirmation of Alzheimer’s disease, and in the more progressed dementia stages where impairments are more overt.

Where documented in the research literature, changes in features of speech in Alzheimer’s disease (including for example, pronoun rate, speech rate, dysfluencies, or partial words), are often manually scored,^15,16^ More recently, speech features have been automatically extracted via natural language processing methods,^17–20^ some of which have been found to correlate with CSF phosphorylated tau biomarkers.^17^ Although individual speech features typically have limited predictive value on their own, they are usually combined via simple machine learning methods to deliver good predictive value for MCI or Alzheimer’s dementia.^12^

More recent methodologies use a data-driven approach to learn patterns directly from raw audio and transcript speech data. Deep learning methods can decompose disease signatures from this highly dimensional data, to identify cognitive processes that are not directly observable, and exploit information from interactions among low level features. Previous approaches have used Transformer-based models pre-trained on extremely large data corpora, to capture a range of linguistic variables. There are two common approaches to using pre-trained Transformer models to make predictions on downstream tasks: attaching a prediction head to a later layer of the Transformer and fine-tuning the entire network,^21^ or using the output at a later layer as a fixed feature extractor and using these features as input to a separate model.^22^ The fine-tuning approach is typical for larger datasets (i.e. thousands of examples) but can be unstable on the small datasets that are typical of clinical studies. Where extracted features are fed into a separate model this poses a different limitation, since the underlying Transformer models have been pre-trained to understand language in general, rather than the specific patterns that change with disease pathology.

For the current paper, we hypothesize that the combination of a sensitive speech task and model architecture could form the basis of speech biomarkers more sensitive to early disease stages. In the current study, speech data are elicited from an automatically administered story recall task, the automatic story recall task (ASRT).^23^ As well as being sensitive to episodic memory impairments, evidence suggests that speech produced during narrative discourse tasks elicit more content rich and varied speech,^24^ and show better differentiation of early Alzheimer’s than other speech sampling strategies.^25^ Further, direct comparison of a story source text with the spoken recall allows for the identification of paraphrases and repetitions, tracking of insertions such as filled pauses or commentary, omissions, or changes in the order or content of the story.

We use ParaBLEU,^26^ a state-of-the-art model optimized for text-pair comparison, allowing direct comparison of source texts and retellings. The model has been trained on a large corpus of text-pairs to evaluate their similarity, which requires the model to understand general linguistic patterns and the ability to compare one text to another. This provides the model with strong inductive biases for evaluating responses on the ASRT.^26^ We evaluate the performance of this model with digitally captured and analysed speech data from the ASRT system (task and model combined), to identify language biomarkers to form the basis of a binary classifier for amyloid positivity and/or cognitive impairment. We compare an automated analysis pipeline with automatic transcription, to a pipeline where speech data are manually transcribed prior to analysis. Furthermore, we complete simulation analysis to examine potential benefits of the ASRT artificial intelligence (AI) system for facilitating MCI referral, and enriching samples for amyloid positivity prior to PET scan. We present results from combined sister studies conducted in the UK and the US.

## Materials and methods

### Study design

AMYPRED-UK and AMYPRED-US studies (clinicaltrials.gov registration NCT04828122, NCT04928976) are prospective studies with data collection planned before the ASRT system index test was performed. The studies used a 2 × 2 cross-sectional design, combining amyloid status (Aβ+ and Aβ) and clinical status (CU and MCI/mild Alzheimer’s disease). Binary reference standards, based on prior clinical trial allocation for Aβ positivity and clinical status were established prior to recruitment into the study. Index test results were therefore not available to the assessors of the reference standard. Primary outcomes were assessed using tournament leave-pair-out cross-validation analysis,^27^ a form of cross-validation used to estimate the model performance on unseen data.

### Participants

Sister studies in two distinct geographical locations were completed: the UK (three sites: London/Guildford, Plymouth, and Birmingham), and the USA (one site: Santa Ana, California).

Potential participants were a convenience sample recruited from trial participant registries between November 2020 and August 2021. Participants were approached if they had confirmed amyloid biomarker status, having undergone a prior Aβ PET scan or CSF test (confirmed Aβ− within 30 months or Aβ+ within 60 months), and if they were CU or diagnosed with MCI in the previous 5 years. In the UK study, participants diagnosed with mild Alzheimer’s disease in the last 5 years were also included. MCI due to Alzheimer's disease and mild Alzheimer’s disease diagnoses were made following National Institute of Aging-Alzheimer’s Association core clinical criteria.^28^

Potential participants were screened via video conferencing (AMYPRED-UK) or in-person (AMYPRED-US), during which the Mini-Mental State Exam (MMSE)^29^ was administered. Inclusion criteria comprised: age 50–85; MMSE raw score of 23–30 for participants with MCI/mild Alzheimer’s Disease, 26–30 for CU; clinical diagnosis made in previous 5 years for participants with MCI/mild Alzheimer’s Disease; English as a first language; availability of a study partner to support completing the Clinical Dementia Rating scale (CDR) semi-structured interview^30^; ability to use and access a smartphone (Android 7 or above or iOS 11 or above), for a fully remote component of the study reported elsewhere.^23^ UK participants required access to the internet on a personal computer, notebook, or tablet supporting audio and video recording for telemedicine appointments. Supported operating systems and internet browser software are provided in the Supplementary methods.

Exclusions comprised current diagnosis of general anxiety disorder; 6-month history of unstable psychiatric illness; history of stroke within the past 2 years; or transient ischaemic attack or unexplained loss of consciousness in the last 12 months. Participants taking medications for Alzheimer’s disease symptoms were required to be on a stable dose for at least 8 weeks. Participants with a current diagnosis (UK study) or a 2-year history of Major Depressive Disorder (US study) were excluded.

### Procedure

Participants completed all assessments with a trained psychometrician via a secure Zoom link (UK study) or in-clinic (US study). For UK study participants, remote assessments were recorded via Zoom after disabling echo cancellation and audio-enhancing features. US assessments were recorded using either a Sony PCM A10 dictaphone or an iPhone 12. Audio recordings were uploaded after each assessment and transferred to Novoic’s servers.

### Clinical assessments

Participants underwent a cognitive and clinical test battery. The full test battery is detailed in Supplementary Table 1 alongside modifications to enable remote assessments for UK participants. Assessments relevant to the current analyses are described below.

The ASRT is an automatically administered story recall task with pre-recorded instructions and stimuli. The ASRT has multiple parallel variants, balanced for linguistic and discourse metrics.^23^ ASRT stories, equivalent in structure, but with differences in names and locations tailored to UK- and US-based locations and landmarks were used. Three long ASRT stories were presented consecutively. Participants were asked to retell each story in as much detail as they could remember both immediately after hearing each story (immediate recall), and again in the same order, after completing all immediate recall trials (delayed recall).

Cognitive tests contributing to the Preclinical Alzheimer’s cognitive composite with semantic processing (PACC5) were administered and mean *z*-score was calculated as previously described.^31^ The composite includes summary scores from five measures: (i) the MMSE,^29^ a global cognitive screening test; (ii) the Logical Memory Delayed Recall,^8,32^ a delayed story recall test; (iii) Digit-Symbol Coding,^33^ a symbol substitution test; (iv) the sum of free and total recall from the Free and Cued Selective Reminding Test,^34^ a multimodal associative memory test; and (v) Category Fluency (animals, vegetables, fruits), a semantic memory test.

The CDR^30^ is a subjectively rated global clinical staging instrument that involves discussions with the participant and informant using a semi-structured interview format. The test was completed by experienced research staff and scored to deliver the Global CDR Score (CDR-G).

In the US study, where participants had completed PACC5 or CDR assessments within 1 month prior to the study visit, tests were not re-administered but the recent historical test results were used.

### Sample size determination

Power calculations completed using the pROC package in R. Prior work has described a threshold of area under the curve (AUC) ≥ 0.75 as being minimally clinically useful.^35^ With significance level set at 0.05, this AUC would be detectable with 99% power for samples of *n* = 50 individuals in each group.

### Outcome measures

Key ASRT system outcomes included the AI-based index test result from speech data identifying: (i) Aβ positivity in the full sample; (ii) MCI in the full sample; (iii) Aβ positivity in MCI/mild Alzheimer’s disease; (iv) Aβ positivity in the CU subsample. Diagnostic accuracy was established through comparison with PET or CSF Aβ status and clinical diagnosis established in prior recent trials. Automatically transcribed ASRTs were the primary measures of interest. Secondary analysis examined data from manually transcribed ASRTs, to identify any change in test accuracy with transcription automation.

### Ethics statement

Informed consent was obtained by qualified staff, at the study site (US sites) or electronically in accordance with HRA guidelines (UK sites). The research was approved by the Institutional Review Boards in the relevant research authorities (UK Research Ethics Committee reference: 20/WM/0116; US Institutional Review Board reference: 8460-JGDuffy).

### Statistical methods

#### Overview of the ASRT system

The ASRT system evaluates story recall as a combination of input pairs, capturing both episodic memory function and linguistic aspects of speech differences between the story source text and spoken recall. The ASRT system was based on the ‘edit encoder’ of the ParaBLEU model, a state-of-the-art machine learning model for text-pair evaluation, which is described in detail in Weston *et al*.^26^ ParaBLEU was adapted for use with the ASRTs using a pre-trained Longformer^36^ model to accommodate longer texts, rather than the RoBERTa^37^ model described in the original publication. Differing from the standard setup, the model was pre-trained with longer text-pair examples from the ParaCorpus^26^ dataset to mirror the length of source-recall pairs from the ASRT, and without the entailment component of the loss function as entailment labels were unavailable for the longer text-pair pre-training dataset. Pre-training was carried out via masked language modelling and autoregressive causal language modelling.

Given two input texts, the edit encoder outputs a vector-based representation encoding the abstract, generalized patterns that differ between them.

#### ASRT system application

Responses were transcribed using Google’s Speech-to-Text^38^ automatic speech recognition system, and also manually following a standardized procedure, and including transcription of commentary, filled pauses and partial words. Analyses were completed in Python and the machine learning package PyTorch. Participants who did not complete ASRT assessments were excluded from onward analysis. The word error rate (WER) of the automatic transcript was calculated using the HuggingFace package^39^ as the average number of errors per manual transcript word. This was calculated after removing punctuation and setting all text characters to lower case, and removing filled pauses and partial words from transcripts prior to comparison.

ParaBLEU was used to derive six vectors for each story, based on the following non-redundant combinations of input pairs: (i) source (original story text) → immediate recall; (ii) immediate recall → source, (iii) source → delayed recall; (iv) delayed recall → source; (v) immediate recall → delayed recall; and (vi) delayed recall → immediate recall. These vectors were averaged to produce one vector for each story in a triplet, and used to train and test predictions of pairs of labels (MCI/mild Alzheimer’s disease or CU; Aβ+ or Aβ−) via logistic regression with the sklearn package in Python. Analysis was completed using tournament leave-pair-out cross-validation analysis. Reference standards (MCI and Aβ labels) were available for training but not for test data. Research has shown that leave-pair-out cross-validation has robust performance relative to other cross-validation approaches, and limited bias.^27^ In tournament leave-pair-out cross-validation analysis, every possible pair of data points is held out in turn while the model is trained using all other data points. The AUC estimate is calculated by ranking the data points according to the model’s predictions. The training set for each test fold comprised all ASRTs from all participants not in the test set. In each fold, the predictions for each recall were ensembled by simple averaging to make participant-level predictions.

#### Clinical and biomarker discrimination of models

Participant-level predictions were used to create a ranking for receiver operating characteristic (ROC) curve analysis. Two comparison models were generated (i) a demographic comparison (age, sex and years of education) and (ii) the PACC5 *z*-score. For three participants, missing data for years in education were replaced with the group median. For 23 participants, one or more PACC5 subtests were not available and PACC5 performance was estimated as the mean *z*-score of their available PACC5 test *z*-scores.

The demographic model was analysed using an identical setup to the models trained on top of the ParaBLEU output vectors. PACC5, for which the input was a single score, was analysed as a logistic regression model within the tournament leave-pair-out framework using the score directly.

Predictions were assessed by the AUC, with accompanying 95% confidence intervals (95%CIs); and sensitivity, specificity and Cohen’s kappa at Youden’s index for the test result in comparison with reference standards. Statistical significance of differences between AUCs, comparing the predictions ASRT system with demographics and PACC5 results, and 95%CIs for AUCs were computed using DeLong’s method.^40^

#### Screening simulation

Screening for MCI and amyloid positivity was simulated in a hypothetical age 65+ sample (*n* = 1000) with proportional representation of each age group representative of the US population,^41^ and MCI prevalence estimates by age from prior meta-analysis.^42^ Prevalence estimates of amyloid positivity by age, and in MCI and CU individuals were also taken from prior meta-analyses.^42,43^ The ASRT system’s sensitivity and specificity within the full sample was determined at Youden’s index, and compared with the MMSE for detecting MCI in prior meta-analysis (pooled sensitivity = 62.7% and specificity = 63.3%).^44^ Furthermore, following methods described by Keshavan *et al*.,^45^ the proportion of PET scans required and the number of participants recruited during pre-screening with the ASRT system was modelled compared with routine PET scanning to deliver a pre-specified sample size.^43^ Simulation methods are detailed in Supplementary methods.

## Results

### Participants

**Figure 1:**
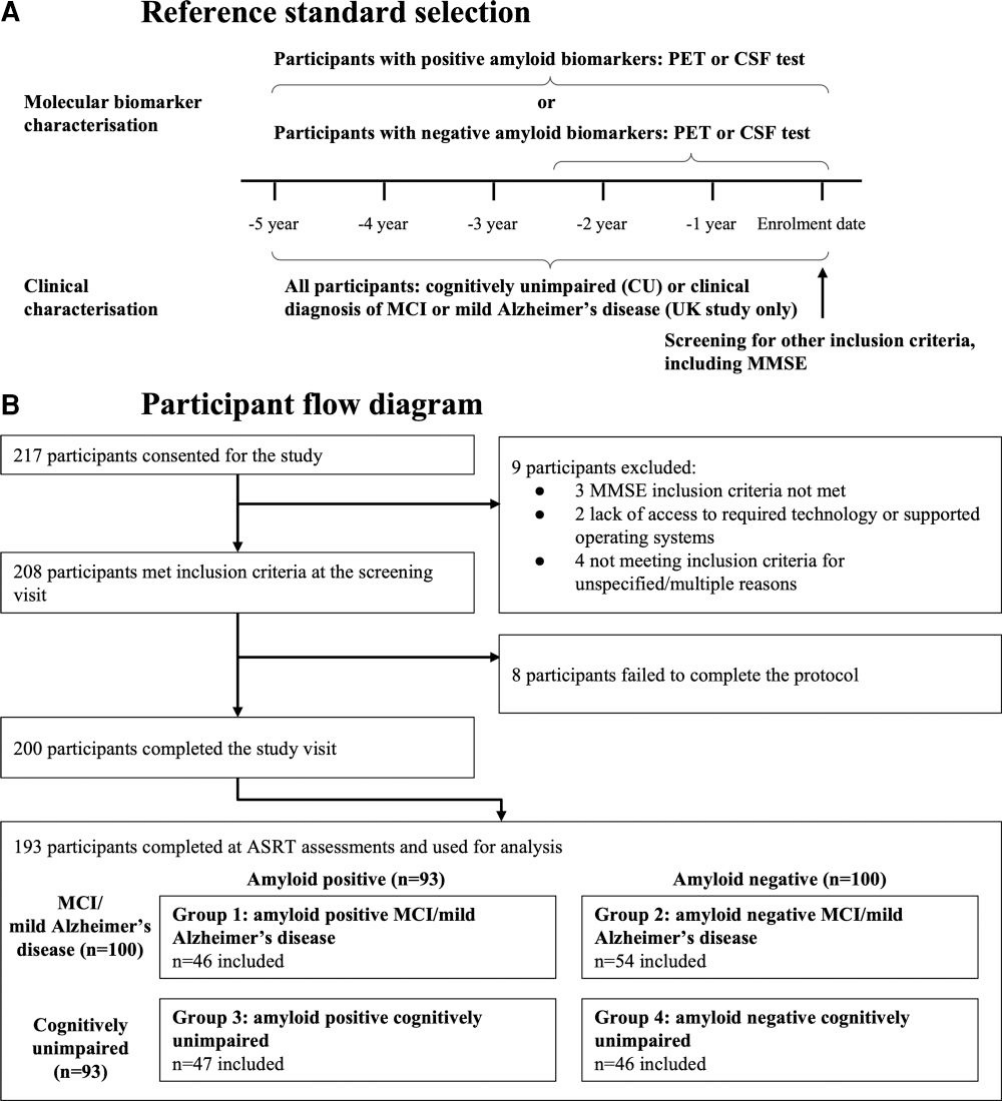
**Participant selection.** (**A**) Participant inclusion criteria: participants were included based on prior amyloid status and clinical diagnosis confirmation. (**B**) Participant flow diagram, documenting exclusions, and dropouts during study recruitment. MCI, mild cognitive impairment; AD, Alzheimer’s disease; MMSE, Mini-Mental State Exam; *n*, number.

Two hundred participants completed the study visit (106/200 MCI/Mild Alzheimer’s disease, and 94/200 CU)—see Fig. 1. Aβ status was confirmed in 88% by PET scan (176/200), 7.5% via CSF (15/200). For 4.5% (9/200), amyloid positivity source information was unavailable. The MCI/mild Alzheimer’s disease participant group comprised primarily MCI participants, with 13 individuals (12.3%) having a diagnosis of mild Alzheimer’s disease.

ASRT assessment recordings were completed by 96.5% (193/200). Six of the seven participants with missing data were in the MCI/mild Alzheimer’s disease group. Participants who did not complete ASRT assessment recordings had lower MMSE scores (*r* = −0.16, *P* = 0.03), but did not differ from the remainder of the group with respect to Global CDR scores (*r* = −0.12, *P* = 0.10), age (*r* = −0.06, *P* = 0.40), years in education (*r* = −0.11, *P* = 0.13), Aβ+/Aβ− ratio (Fishers exact test, *P* = 0.64), or male/female ratio (Fishers exact test, *P* = 0.99).

Two participants became distressed during cognitive assessments. One aborted their participation in the study and was excluded from further analyses; the other participant partially completed assessments but was happy and able to continue and was included. No other adverse events were reported.

Demographics in the sample completing ASRTs and included in analyses are shown in Table 1. Groups were overall well matched for most demographic variables. Age at assessment differed significantly between Aβ+ and Aβ− biomarker groups in the full sample. In subgroup analyses, age differences were seen between Groups 1 and 2 (Aβ+ and Aβ− participants with MCI/mild Alzheimer’s disease), and Groups 1 and 4 (MCI/mild Alzheimer’s disease Aβ+ and CU Aβ− participants). Demographics separated by AMYPRED-UK and AMYPRED-US studies are provided in Supplementary Tables 2 and 3.

**Table 1:** Participant demographic and clinical characteristics: UK and US samples

	Subgroup analyses					Full sample analyses					
	Group 1: (*N* = 46)	Group 2: (*N* = 54)	Group 3: (*N* = 47)	Group 4: (*N* = 46)	*P*-value	Clinical group			Biomarker group		
						CU (*N* = 93)	MCI/mild Alzheimer’s disease (*N* = 100)	*P*-value	Amyloid beta negative (*N* = 100)	Amyloid beta positive (*N* = 93)	*P*-value
Amyloid beta positive/negative (*N*)	Positive	Negative	Positive	Negative	—	47/46	46/54	0.53	Negative	Positive	—
MCI/CU group (*N*)	MCI/mild Alzheimer’s disease	MCI/mild Alzheimer’s disease	CU	CU	—	CU	MCI	—	54/46	46/47	0.53
Female/male (*N*)	21/25	34/20	27/20	28/18	0.32	55/38	55/45	0.56	62/38	48/45	0.15
Years in education, mean (SD)	15.22 (3.09)	14.92 (2.81)	15.06 (3.53)	15.75 (3.08)	0.57	15.40 (3.32)	15.06 (2.93)	0.34	15.31 (2.95)	15.14 (3.31)	0.34
Age, mean (SD)	72.72^A^ (5.95)	68.65^B^ (7.45)	71.43^C^ (4.77)	69.41^D^ (4.10)	0.001^AB, AD^	70.43 (4.54)	70.52 (7.07)	0.35	69.00 (6.12)	72.06 (5.40)	<0.001
MMSE, mean (SD)	26.64^A^ (2.18)	26.89^B^ (2.16)	28.77^C^ (1.43)	28.78^D^ (1.07)	<0.001^AC, AD, BC, BD^	28.77 (1.26)	26.78 (2.16)	<0.001	27.76 (1.98)	27.73 (2.11)	0.87
CDR-G, mean (SD)	0.57^A^ (0.17)	0.54^B^ (0.17)	0.12^C^ (0.22)	0.09^D^ (0.19)	<0.001^AC, AD, BC, BD^	0.10 (0.20)	0.55 (0.17)	<0.001	0.33 (0.29)	0.34 (0.30)	0.88

Demographic and clinical characteristics shown by research Groupings 1–4, and summary statistics for participants characterized by clinical diagnostic or biomarker profiles. MCI, mild cognitive impairment; CU, cognitively unimpaired; *N*, number; SD, standard deviation. Group 1, amyloid beta positive MCI/mild Alzheimer’s disease; Group 2, amyloid beta negative MCI/mild Alzheimer’s disease; Group 3, amyloid beta positive cognitively unimpaired; Group 4, amyloid beta negative cognitively unimpaired; MMSE, Mini-Mental State Exam; CDR-G, Global Clinical Dementia Rating Scale Score. A, B, C, D correspond to contrasts relating to p-values within row, e.g. *P*-value for AC is the comparison between data from cells A and C in the same row.

### ASRT system application

**Figure 2:**
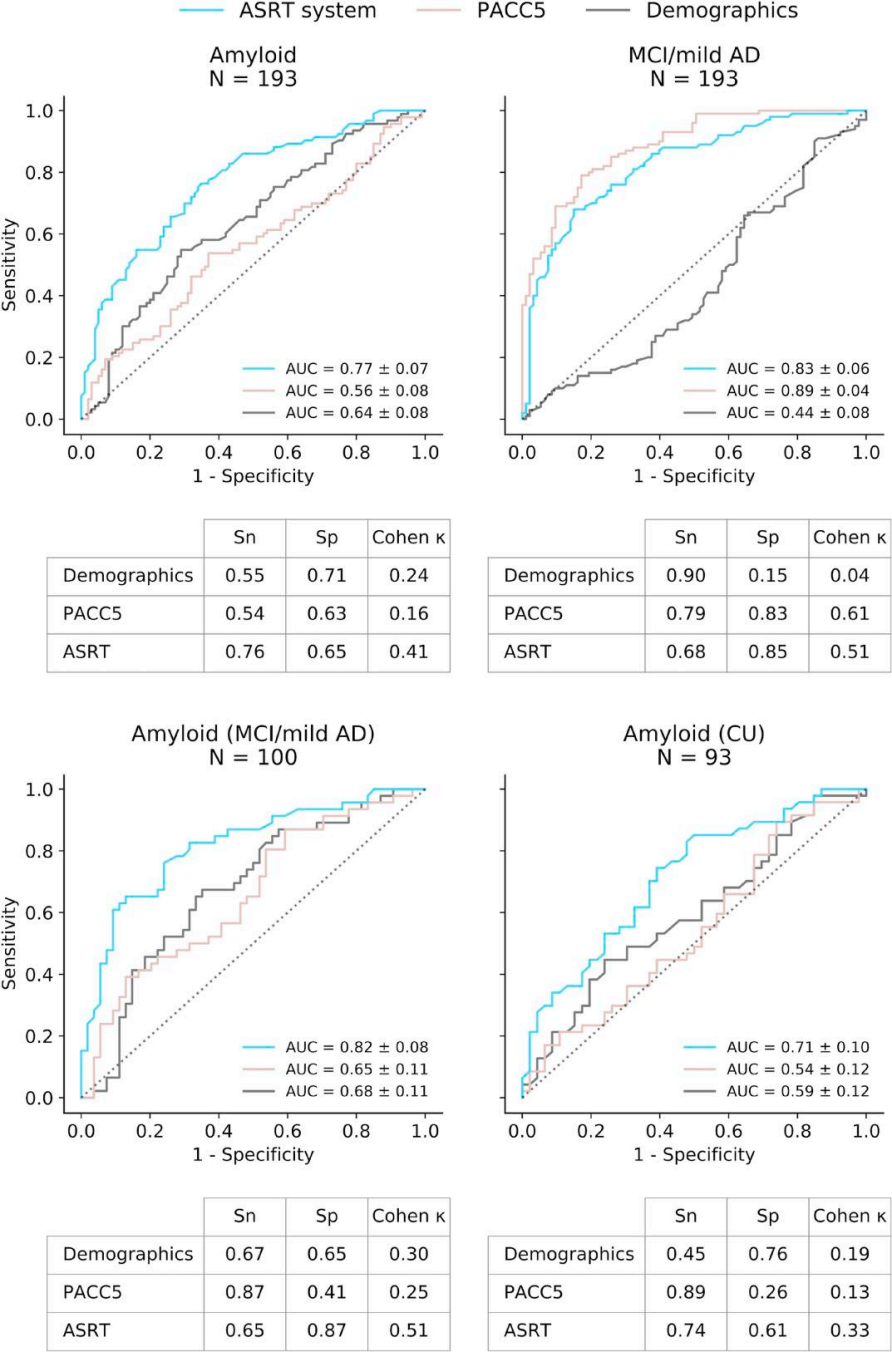
**ROC curves for the ASRT system and comparison models.** AUCs and 95% confidence intervals for the classifiers predicting: (**A**) amyloid positivity and (**B**) mild cognitive impairment (MCI)/mild Alzheimer’s disease in the full sample. Subsample comparisons of classifier performance predicting (**C**) amyloid positivity within the MCI/mild Alzheimer’s disease; and (**D**) amyloid positivity in the CU sample. The table below each figure provides sensitivity (Sn) and specificity (Sp) at Youden’s index and Cohen’s kappa (Cohen K). The reference test was biomarker confirmation from PET or CSF for **A**, **C**, and **D**. Reference test was clinical diagnosis for **B**. The demographic comparison includes age, sex and education level. AD, Alzheimer’s disease; ASRT, automatic story recall test; PACC5, preclinical Alzheimer’s cognitive composite with semantic processing; ROC, receiver operator characteristic; AUC, area under the curve.

The ASRT task yielded on average over 6 (6.56) min of speech per participant. The ASRT system area under the ROC curve (AUC) of our primary endpoint, Aβ classification in the full sample was 0.77 (95%CIs ±0.07) (Fig. 2A), above chance (*z* = 7.89, *P* < 0.001) and with significantly better prediction than demographics (*z* = 2.69, *P* < 0.01) and PACC5 (*z* = 4.37, *P* < 0.001). The AUC for predicting Aβ classification within the MCI sample (Fig. 2C) was even higher at AUC = 0.82 (95%CIs ±0.08), above chance (*z* = 7.27, *P* < 0.001) and superior again to demographics (*z* = 2.27, *P* = 0.02) and PACC5 (*z* = 2.64, *P* = 0.008). In the CU subsample (Fig. 2D), the ASRT system AUC for Aβ detection was 0.71 ±0.10, above chance (*z* = 4.03, *P* < 0.001) and better than PACC5 (*z* = 2.34, *P* = 0.02), but not superior to demographics (*z* = 1.63, *P* = 0.10).

MCI classification in the full sample using the ASRT system in the full sample yielded an AUC of 0.83 (95%CIs ±0.06) (Fig. 2B), significantly better than chance (*z* = 11.02, *P* < 0.001) and demographics (*z* = 8.01, *P* < 0.001) but not better than the PACC5, which showed modestly better performance than the ASRT system (*z* = −2.20, *P* = 0.03).

Contrast of results from manual and automatic transcription is shown in Supplementary Fig. 1. Manual and automatically transcribed models were broadly overlapping. The pattern of differences with comparison models was the same, with the exception of the model for detecting Aβ in CU participants, where manually transcribed data had a modestly lower AUC (0.65, 95%CIs ±0.11), which although remaining better than random (*z* = 2.61, *P* = 0.009), did not perform better than PACC5 (*z* = 1.41, *P* = 0.16), or demographics (*z* = 0.82, *P* = 0.41). AUCs generated for manually and automatically transcribed data did not differ (MCI full sample: *z* = 0.39, *P* = 0.72; amyloid full sample *z* = 1.03, *P* = 0.30; amyloid in MCI: *z* = 0.36, *P* = 0.71; amyloid in CU: *z* = 1.91, *P* = 0.06). Average WER across participant recordings for automatic transcripts when compared with manual transcripts, was 0.16.

### Screening simulation

In a simulated population sample age 65+ (MCI prevalence 15.4%) screening for MCI in primary care using the ASRT system is estimated to modestly improve detection of individuals with MCI by 8.5% and reduce false positives by 59.1% in comparison with screening with the MMSE. This represents an increased positive predictive value from 23.7 to 45.3% and negative predictive value from 90.3 to 93.6%.

The potential benefit of speech-based Aβ screening prior to PET scan was examined in a simulated population aged 65–85. In individuals with MCI (overall Aβ+ prevalence estimated at 55.9%) for a pre-specified sample size of PET Aβ+ individuals, screening using the ASRT system prior to PET scanning was estimated to reduce the number of PET scans required by 35.3%. This reduction is dependent on prevalence and therefore also age,^43^ with the greatest benefit of screening seen for younger individuals. In CU individuals aged 65–85 (Aβ+ prevalence 24.9%), screening using the ASRT system could reduce the number of PET scans required by 35.5%.

## Discussion

The current study presents the ASRT system, an automatically administered and analysed screening test, analysed with an advanced AI system to predict Aβ positivity (AUC = 0.77 ±0.07) and MCI (AUC = 0.83 ±0.06). Aβ positivity is detectable in speech in individuals with MCI/mild Alzheimer’s disease (AUC = 0.82 ±0.08), and in otherwise CU individuals (AUC = 0.71 ±0.10). The results reveal changes in speech occurring at the earliest stages in the disease. Further, we find similar results for automatic and manually transcribed data, despite moderate levels of transcription errors. This indicates that at the level noted in the current study, transcription errors do not significantly impact the sensitivity of the ASRT system.

The binary classification ability reported here is similar to those previously reported from other studies identifying MCI/mild AD from speech assessment in the literature.^10^ For detecting MCI, the ASRT system also performs similarly to a range of other available traditional cognitive tests, as shown in prior meta-analysis (AUC 0.70–0.94, mean AUC across tests evaluated AUC = 0.81).^46^ In the current study, the ASRT system is superseded by the PACC5 cognitive composite for detecting MCI.

In the current study the ASRT system is superior to the PACC5 for detecting Aβ positivity. Research indicates that traditional cognitive tests and cognitive composites, although sensitive to cognitive decline, on their own show more modest differentiation between amyloid positive and negative individuals,^47–49^ reflected also in the current results. Together, this indicates that subtle differences in which the way spoken cognitive tasks are performed may be more predictive of amyloid positivity than more standard measures of recall or response accuracy typically obtained in cognitive assessment.

A positive prediction of amyloid from speech data alone has not been reported before, although amyloid sensitivity has been shown for certain cognitive tests,^49^ and prior work shows sensitivity of speech measures to phosphorylated tau but not beta amyloid biomarkers as measured via CSF test.^17^ The greater sensitivity to amyloid identified in the current study, as compared with prior research, could be attributed to a combination of factors. First, the study design allowed for combined and separate evaluation of changes in speech associated with MCI, and underlying biomarker profiles. Second, the task used is sensitive to episodic memory impairments commonly seen in early-stage Alzheimer’s disease, and is likely to generate more linguistically varied responses than other common speech tasks.^24^ Third, the design of the model as a text comparisons system allows for the evaluation of the participant response relative to a source text, identifying not just the frequency of key linguistic differences, but also embedding these changes within context in which they occur.

A recent report from the Lancet Commission indicates that 12 modifiable risk factors account for around 40% of worldwide dementia cases, which could theoretically be prevented or delayed.^50^ Aduhelm, the first disease-modifying treatment for Alzheimer’s disease was approved by the FDA in June 2021 through its Accelerated Approval pathway using brain amyloid load as a surrogate endpoint. The approval of Aβ as a surrogate for the treatment of Alzheimer disease may open a ‘floodgate’ of amyloid-targeting drugs.^51^ Treatments will primarily have been tested in patients with MCI or mild Alzheimer’s disease with elevated amyloid biomarkers and will likely be indicated in these populations.

Effective screening and early detection of MCI/mild Alzheimer’s disease, biomarker-positive individuals could help quickly and appropriately identify patients for clinical trials and/or approved treatments, potentially reducing interim cognitive deterioration. Affordable and accessible testing to direct the appropriate patient population to further assessments and treatment is a key to controlling healthcare system costs of these drugs. Blood-based testing for Alzheimer’s disease holds promise for widespread and lower cost diagnostic testing, and is expected to approach clinical use in a few years.^52^ However, research to date indicates that blood-based biomarkers do not differentiate clinical stages of the disease well,^53^ which indicates that even with further improvement in the sensitivity and consistency of blood-based testing a continued need for cognitive and clinical assessment will remain. Furthermore, blood-based testing remains invasive, requires in-person assessment, and usually has a turnaround time of days to weeks, whereas speech-based diagnostic assessment can be completed non-invasively, remotely and with instantaneous and automatic generation of results. Prior work using the ASRT system administered in a remote setting shows that participants report the application to be easy to use and the tasks broadly interesting.^23^

The ASRT system requires no trained personnel or specialist equipment, and could improve efficiency of screening for MCI and mild Alzheimer’s disease, making it possible for patients and clinicians to engage in more routine cognitive monitoring or health checking. Furthermore, screening for amyloid positivity in MCI may help to identify whether patients are likely at risk of disease progression. This can help to support risk reduction approaches, and initial screening for suitability of approved disease-modifying treatments.

Finally, The ASRT system can help to reduce costs in clinical trials by enriching recruited samples. To obtain a pre-specified sample size of Aβ+ individuals, pre-screening using the ASRT system would require recruitment of a higher number of participants (+53.8% in MCI, and +35.1% in CU participants), but reduce the volume of costly PET scans needed (−35.3% in MCI, and −35.1% in CU individuals).

### Limitations

In the current study, participant recruitment was dependent on the availability of prior amyloid PET and CSF amyloid test results within the past 30 (Aβ−) to 60 months (Aβ+). Since Aβ positivity increases with age,^43^ conversion may have occurred for some participants in the interim period. CSF and PET Aβ positivity are differentially associated with cognitive decline and may be optimally sensitive at different disease stages.^54^ Variation in biomarker and diagnostic criteria (between trials where participants were recruited from) is likely to have introduced increased variability in our diagnostic reference standards. Even a small number of false labels can impact training of AI systems. Improvements in model performance could be expected with concurrent and consistent reference standards, and with quantitative rather than binary amyloid results.

In the UK, cognitive assessments comprising the PACC5 were completed via telemedicine, which deviates from typical test administration which is carried out in clinic. However, test results shown here are in keeping with prior research administering similar cognitive composites in clinic.^47^ There was also a high level of missing data from PACC5 subtests (in 11.5% of participants) where data collection was cut short due to time limitations or participant fatigue. Averaging *z*-scores across the existing subtests for these participants is likely to provide a reasonable estimate of generalized cognitive ability.

Our demographic baselines included age, sex and education and additional sensitivity for demographic predictors could be gained with the inclusion of family history for Alzheimer’s and Apolipoprotein E (APOE) genotype, which are known risk factors. Similarly, combining the algorithm with other risk factors (e.g. age, APOE genotype) could help to further increase discriminative power. Our analysis was limited to textual analysis of transcribed retellings, and acoustic and temporal features of the voice recording were not evaluated in the current study. Additional sensitivity to cognitive impairment and amyloid positivity could be afforded through the inclusion of this additional information in future analyses.^11^

Our simulation analyses evaluate hypothetical savings in clinician resources and PET scans given the sensitivity and specificity of the ASRT system test results derived in the current study. Although study samples were carefully recruited in a balanced fashion, with no overall differences noted between the groups on key demographic variables, it is not clear to what extent subtle variation in these measures between groups, or other unmeasured demographic imbalances that may impact our models. While the results show promise for reducing referral burden and cost savings when screening for amyloid positivity prior to PET scan, the results require replication and therefore should be interpreted with caution.

Moreover, due to lack of evaluation of ethnic and racial variation of Aβ in the cited meta-analysis used to generate prevalence levels in our simulation,^43^ it is noted that the reported amyloid positivity rates may not be reflective of all racial and ethnic groups.^55^ Similarly, the population under examination here showed limited ethnic or racial diversity (*N* = 193 white, *N* = 3 Asian and *N* = 4 Black or African American), indicating that replication across a broader and more representative range of ethnic and racial backgrounds is required. Participants were also required to have access to and ability to use a smartphone, which may have precluded a subset of individuals from taking part.^56^ While our findings, based on a combined UK and US sample, indicate that the robust results can be achieved across different geographical locations and accents, replication and, ideally, out-of-sample validation in larger, more clinically and demographically heterogeneous samples is now needed.

## Supplementary Material

fcac231_Supplementary_Data

## Data Availability

The data that support the findings of this study are available on reasonable request from the corresponding author. Speech data are not publicly available due to information that could compromise the privacy of research participants.

## Abbreviations

Aβ =: amyloid beta
AI =: artificial intelligence
APOE =: Apolipoprotein E
ASRT =: automatic story recall task
AUC =: area under the curve
CDR =: Clinical Dementia Rating scale
CDR-G =: Clinical Dementia Rating scale global score
CU =: cognitively unimpaired
MCI =: Mild cognitive impairment
MMSE =: Mini-Mental State Exam
*n* =: number
PACC5 =: Preclinical Alzheimer’s cognitive composite with semantic processing
ROC =: receiver operating characteristic
SD =: standard deviation
UK =: United Kingdom
US/USA =: United States of America
95%CIs =: 95% confidence intervals
